# Diagnostically challenging human papillomavirus-associated primary squamous cell carcinoma of the rectum with metastasis in both ovaries: a case report

**DOI:** 10.1186/s13256-020-2348-5

**Published:** 2020-02-14

**Authors:** P. Tschann, D. Lechner, B. Feurstein, B. Abendstein, S. Dertinger, A. Bösl, N. Vitlarov, F. Offner, I. Königsrainer

**Affiliations:** 1 Department of General and Thoracic Surgery, Academic Teaching Hospital Feldkirch, Feldkirch, Austria; 2 Department of Gynaecology, Academic Teaching Hospital Feldkirch, Feldkirch, Austria; 3 Institute for Pathology, Academic Teaching Hospital Feldkirch, Feldkirch, Austria

**Keywords:** Squamous cell carcinoma, Human papillomavirus, Rectum, Metastasis, Ovary

## Abstract

**Introduction:**

Squamous cell carcinomas of the rectum are extremely rare and their pathogenesis is still under debate. Their proper diagnosis and treatment may thus be challenging.

**Case presentation:**

A 52-year-old Caucasian woman was transferred to our department with a history of pelvic pain. Colonoscopy revealed a small tumorous lesion of the upper rectum and an endoscopic biopsy showed infiltration of the rectal mucosa by a squamous cell carcinoma. Afterward, tumorous lesions were found on imaging in both her ovaries. A laparoscopy with adnexectomy and anal mapping was performed and revealed tumor masses of squamous cell carcinoma in both ovaries. Based on the large size of the ovarian tumors and the concurrence of extensive, partly ciliated, macrocystic epithelium in one of the ovaries, a diagnosis of ovarian squamous cell carcinoma arising from a mature teratoma was rendered. However, human papillomavirus genotyping analyses were positive for human papillomavirus-16 in both the rectal tumor and ovarian tumors leading to a final diagnosis of a human papillomavirus-associated rectal squamous cell carcinoma metastatic to both ovaries. Neoadjuvant chemoradiation therapy of her rectum, total mesorectal excision, and hysterectomy were performed followed by adjuvant chemotherapy.

**Conclusion:**

Colorectal squamous cell carcinoma is a rare disease. In cases of colorectal squamous cell carcinoma, metastatic disease at any other location has to be excluded. Human papillomavirus genotyping is essential in this context. Discussion of the treatment strategies should be interdisciplinary and include chemoradiation therapy and radical surgery.

## Introduction

Colorectal squamous cell carcinoma (SCC) as a primary tumor localization is exceedingly rare and represents only 0.1 to 0.25 per 1000 cases of all colorectal cancers [1–4]. The majority of the cases are actually anal SCC with proximal extension into the rectum. These cases are largely attributed to human papillomavirus (HPV) infections [5]. In contrast, in the higher rectum there is no clear evidence for an association with HPV infections but an association with human immunodeficiency virus (HIV) [5–7]. One possible explanation of HPV infection in the higher rectum is circulating HPV deoxiribonucleic acid (DNA) [8]. Anal SCC affects more women than men and typically appears in the sixth decade of life, mostly with pelvic pain and bleeding [4, 9, 10]. Liver and lung are known to be the first metastasized organs, similar to colorectal adenocarcinoma. The ovaries as first afflicted organ is rare, but well described, and should be considered in the diagnostic pathway. Moreover, approximately 4% of women with intestinal cancers have ovarian metastases at some time in the course of their disease and these are misinterpreted as primary ovarian tumors in 3–20% of cases [11].

In the diagnostic work-up of rectal SCC three possibilities have to be considered: (a) a rectal involvement by an anal SCC, (b) an anal SCC-lined rectum fistula, and (c) a metastasis from another primary tumor location [12, 13]. The diagnosis of SCC should include tissue biopsy, endosonographic ultrasound, magnetic resonance imaging (MRI) of the rectum, and computed tomography (CT) scan.

This case shows a rare constellation of a primary SCC in the rectum with metastasis in both ovaries and an association with HPV infection in a HIV-negative patient. Chemoradiation with or without surgery are the main treatment options, depending on tumor localization, patient’s condition, and functional aspects.

## Case presentation

**Fig. 1:**
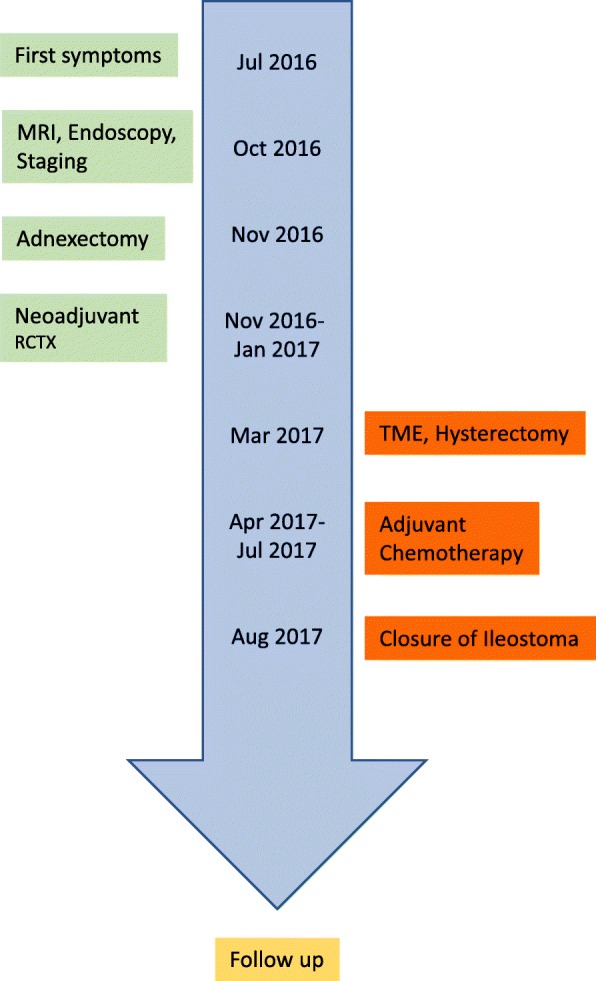
Timeline of patient’s history. *MRI* magnetic resonance imaging, *RCTX* radiochemotherapy, *TME* total mesorectal excision

A 52-year-old Caucasian woman was referred to our department in November 2016 with a history of pelvic pain for more than 3 months. Previous to our consultation she had consulted with an orthopedic physician and received physical therapy. Her family, social, and environmental histories showed no abnormalities. She did not smoke tobacco and was not addicted to alcohol or any drugs. No prior medication was reported. Weight loss or B symptoms were not recorded (her history is shown as a timeline in Fig. 1). At the first clinical presentation, a blood pressure of 140/90 mmHg and a pulse of 100 beats per minute (bpm) were observed. Her physical and neurological examinations were uneventful. Results of laboratory findings are listed in Table 1.

**Table 1:** Laboratory results at first clinical visit

Natrium	136	mmol/l
Potassium	3,8	mmol/l
Calcium	2,41	mmol/l
Creatinin	0,7	mg/dl
GFR-CKD/173m2	101	ml/min
Urea	29	mg/dl
Total Bilirubin	0,2	mg/dl
GPT	34	U/l
GGT	135	U/l
APH	95	U/l
Lipase	62	U/l
CRP	0,04	mg/dl
Quick	>120	%
PTT Actin FS	24,6	Sec.
Leukocyte	8,6	G/l
Erythrocyte	4,09	T/l
Hämoglobin	124	g/l
Hämatokrit	0,37	L/l
MCV	89,5	fl
MCH	30,3	pg
MCHC	339	g/l_
Platelet	319	G/l

*APH* Alkaline phosphatase, *CKD* chronic kidney disease, *CRP* C-reactive protein, *GFR* glomerular filtration rate, *GGT* gamma-glutamyltransferase, *GPT* glutamate-pyruvate transaminase, *MCH* mean corpuscular hemoglobin, *MCHC* mean corpuscular hemoglobin concentration, *MCV* mean corpuscular volume, *PTT* partial thromboplastin time

**Fig. 2:**
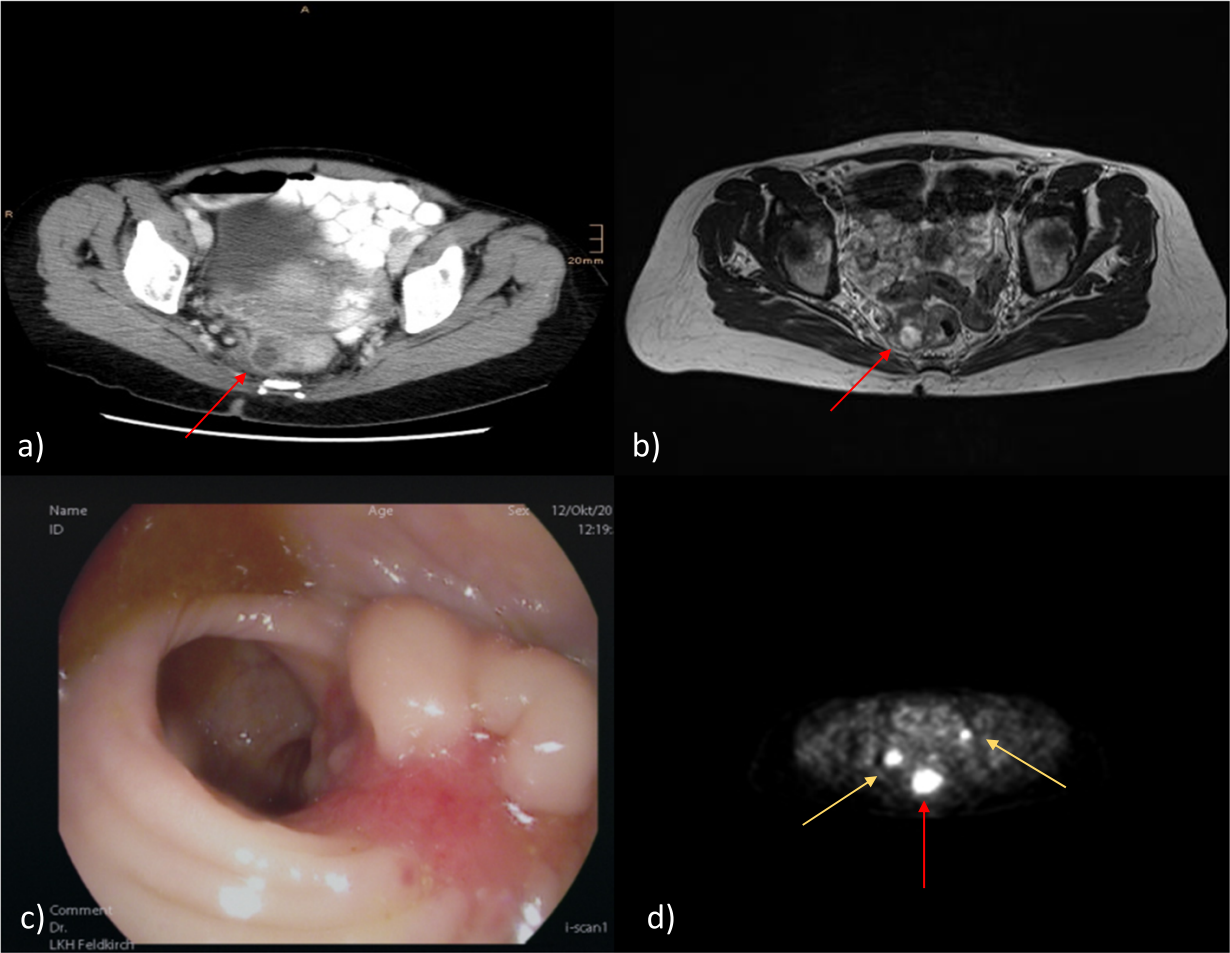
Preoperative imaging. **a** Computed tomography scan. **b** Magnetic resonance imaging axial. **c** Endoscopy. **d** Positron emission tomography scan. In computed tomography scan and magnetic resonance imaging only, primary tumor was clearly detected (*red arrow*). A cystic ovary was described. To exclude foreign metastasis a positron emission tomography scan was performed with an enhancement in both ovaries (*yellow arrows*)

After unsuccessful physical therapy and persistent pelvic discomfort, she was sent for an MRI, where a 30 mm tumorous lesion was found in her middle rectum. A proctoscopy showed a tumorous lesion 7–10 cm from the anal verge (Fig. 2a–d, preoperative staging).

**Fig. 3:**
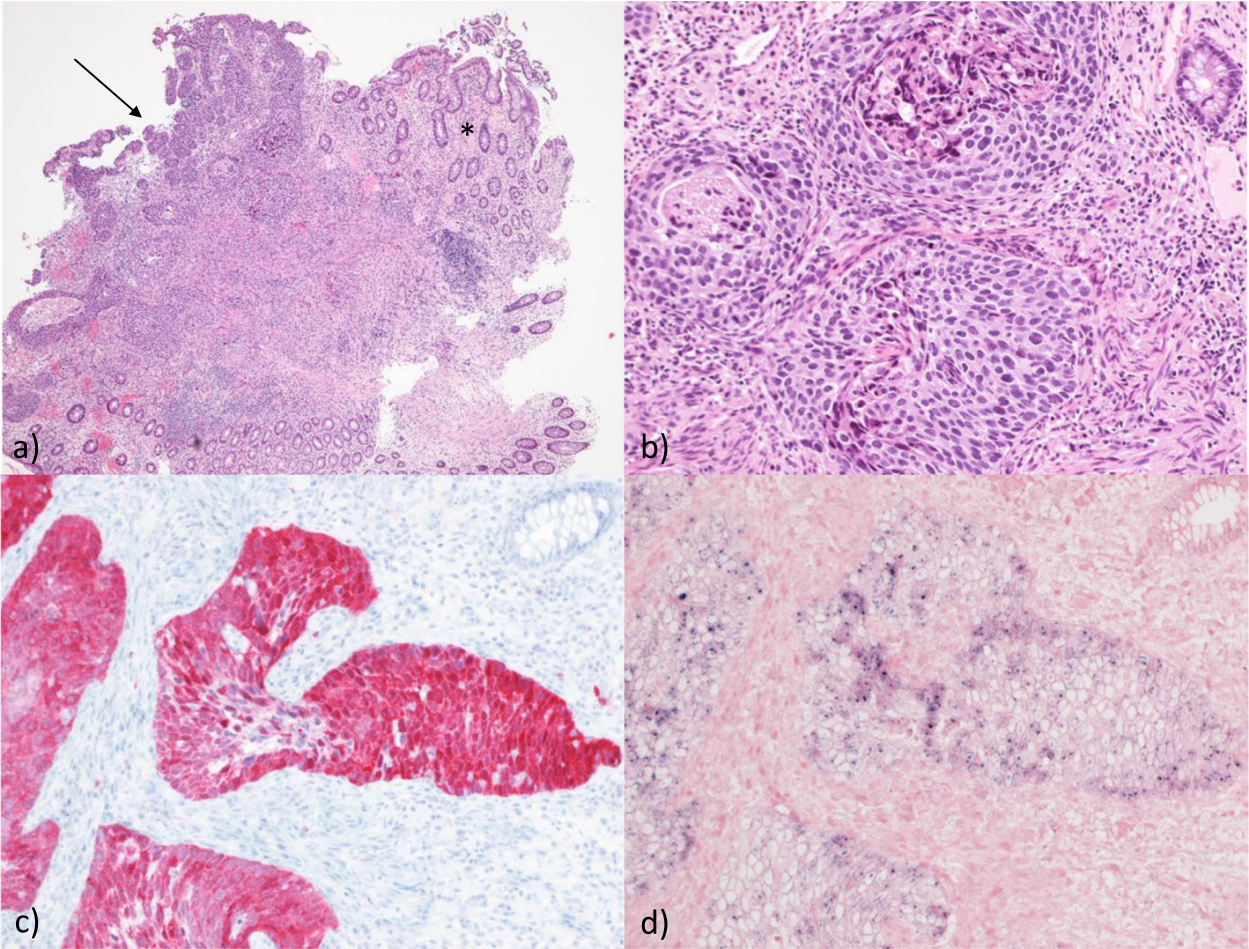
Squamous cell carcinoma of the middle rectum. **a** The tumor involves the mucosal surface and focally displays a tubular growth pattern (*arrow*) resembling the architecture of adjacent crypts of the normal rectum (*arrow*). This pattern may be interpreted as an *in situ* lesion with growth of tumor cells along the basement membranes of the colonic crypts (*). **b** The invasive tumor component in the submucosa is moderately to poorly differentiated. **c** The tumor expresses p16. **d** Shows a positive *in situ* hybridization result when analyzing for high risk human papillomaviruses

The histopathological result of a tissue biopsy was HPV-16 associated SCC (Fig. 3a–d); local staging was performed with endoscopic ultrasound and showed a uT3uN1 stage in the middle rectum. Cancer antigen (CA) 19-9 and carcinoembryonic antigen (CEA) levels were initially elevated (CA 19-9, 42 U/ml; CEA, 6.3 ng/ml); CA 125 was not increased (5 U/ml). A HPV screening of her husband was not performed.

There was no evidence of metastasis in a CT scan of her trunk. However, in positron emission tomography (PET)-CT a mild enhancement in both ovaries was observed.

**Fig. 4:**
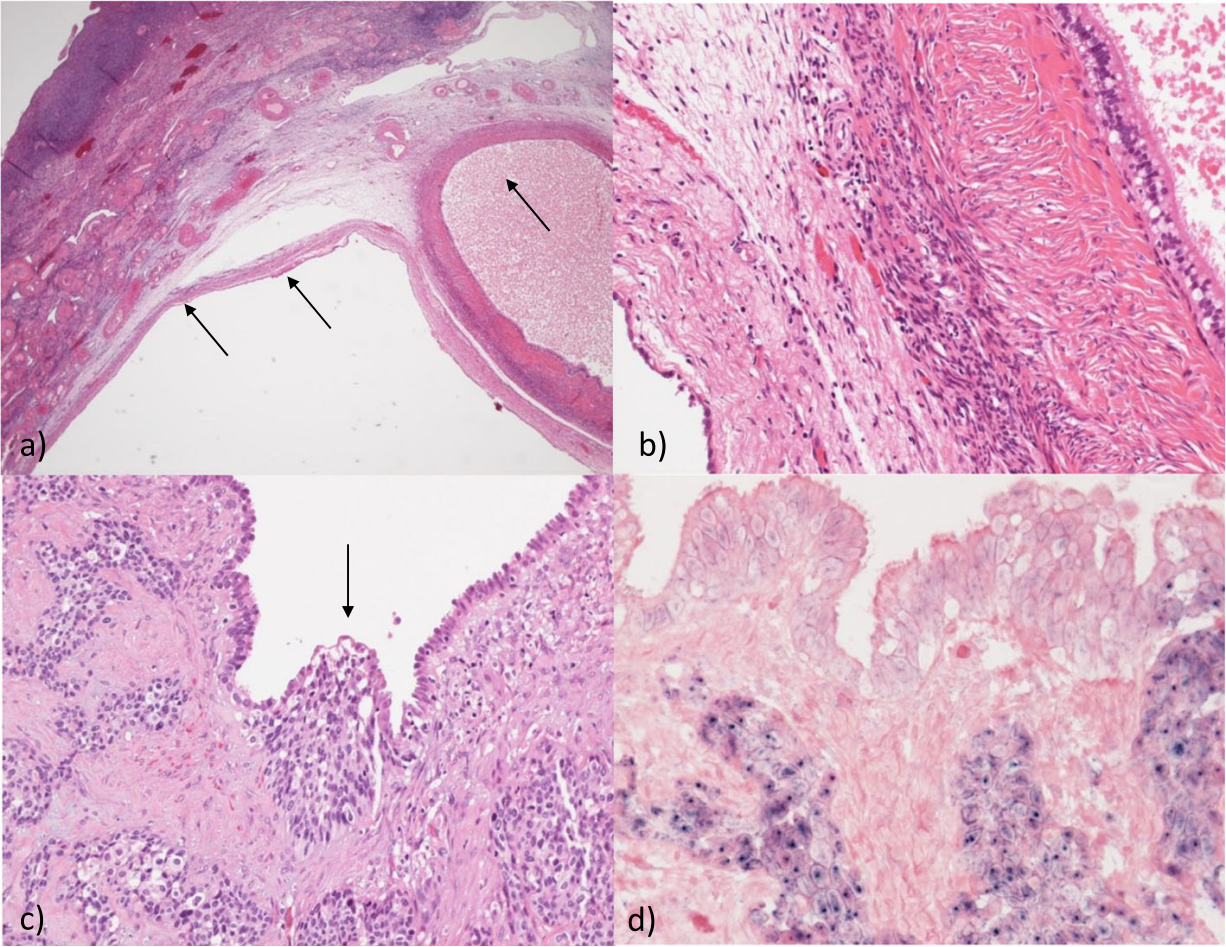
Histologic analyses of the ovaries. Both ovaries showed infiltration by poorly differentiated squamous cell carcinoma. **a**, **b** In the right ovary there were multiple cystic gland-like structures (*arrows*) lined by flat to cuboidal or ciliated epithelium. **c** The glandular epithelium was intimately intermingled with infiltrative clusters of the squamous cell carcinoma (*arrow*) leading to an initial diagnosis of a teratoma with malignant transformation. **d***In situ* hybridization for high risk human papillomaviruses, however, was positive in the squamous cell carcinoma cells and negative in the cystic glandular epithelium, strongly arguing against this interpretation of the findings

**Fig. 5:**
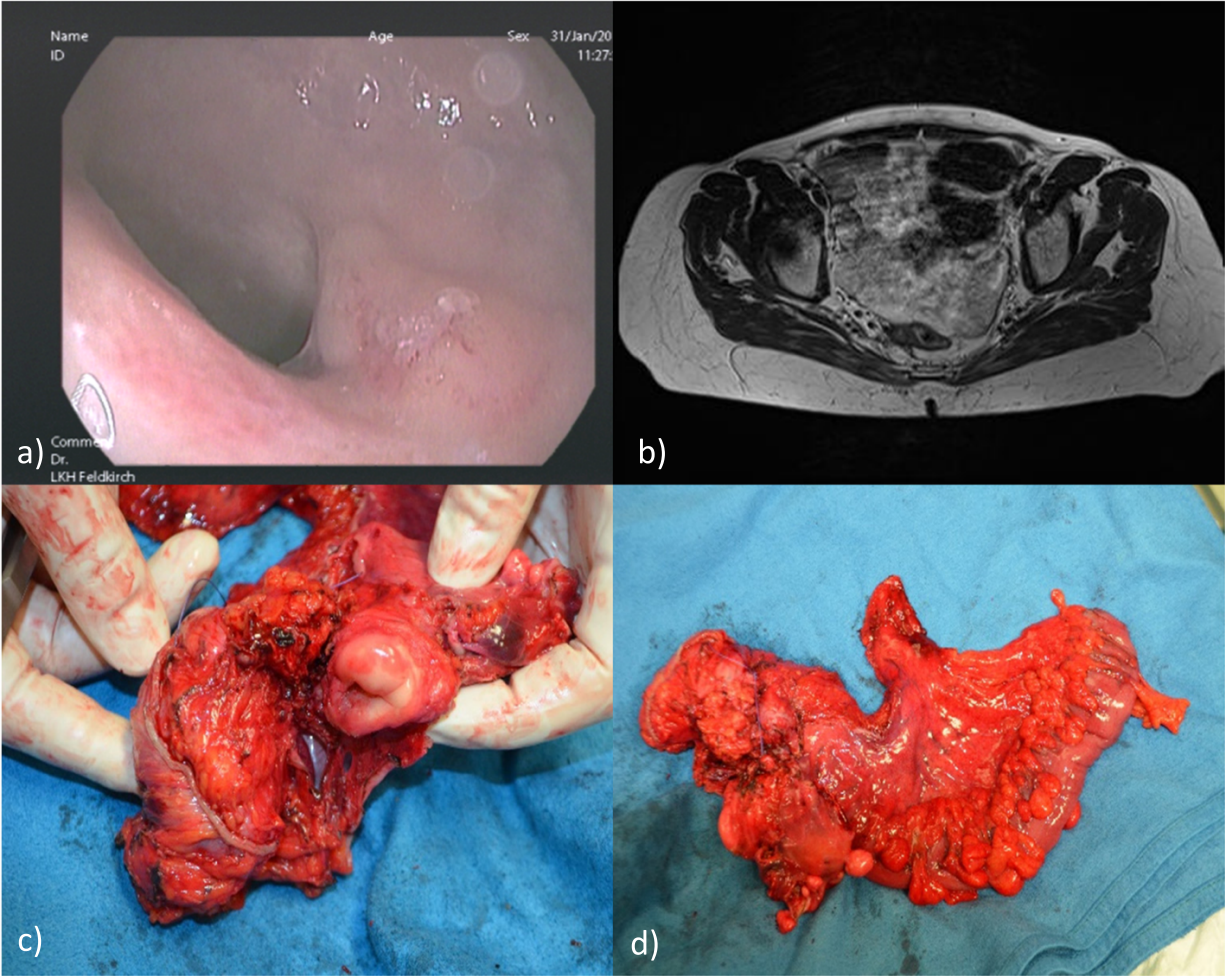
**a**, **b** Endoscopy after chemoradiation therapy with a good clinical and imaging (magnetic resonance imaging) response. On the assumption of a malignant transformed teratoma of the ovary an open total mesorectal excision and hysterectomy en bloc was performed as operative strategy (**c**) and (**d**)

Consequently, a diagnostic laparoscopy, adnexectomy, and anal mapping were performed. Histopathologic analyses of her ovaries revealed double-sided SCCs and in one of her ovaries extensive cystic structures lined by flat cuboidal and ciliated epithelium. Focally, the cystic epithelium was intimately intermingled with the squamous tumor cells and a diagnosis of a mature teratoma with malignant transformation into SCC was rendered (Fig. 4a–d). The anal mapping exhibited a small, barely visible, anal intraepithelial neoplasia (AIN) grade II without any contact with the rectal mucosa and without any evidence of invasion. The immunohistochemical panel was p16 and p53 positive. Based on the pathologist’s reports of the ovaries, the rectal SCC lesion was first interpreted as a metastasis of the malignant ovarian teratoma. Malignant transformation of ovarian teratoma is not that rare (0.17–2%), whereas SCC transformation is common (80%) [14]. Metastasis to the mesorectum of a SCC-transformed teratoma is also reported [15]. However, a two-step approach similar to the treatment of nodal-positive rectal cancer was chosen, that is, preoperative chemoradiation therapy (CRT) of the rectum with 45 Gy and cisplatin, followed by total mesorectal excision (TME) with hysterectomy en bloc and protective loop ileostomy 6 weeks after CRT with a strong endoscopic and radiological response (Fig. 5a–d). On histologic work-up, only a small remainder of SCC was found in her mesorectum and the resection margins were free from disease.

Her postoperative course was uneventful. She underwent adjuvant chemotherapy with cisplatin and vinorelbin. The closure of loop ileostomy was performed after systemic therapy. The follow-up was similar to that for colorectal cancer with clinical examination including the monitoring of CA 19-9 and CEA levels every 3 months in the first year. After 12 months a colonoscopy, CT scan, PET scan, and MRI were performed. Annual pelvic MRI, CT scan, and PET scan were used for follow-up with a disease-free survival of 36 months up to now (11/2016–10/2019). Quality of life and function after TME are adequate.

The course of treatment was similar to rectal cancer. The neoadjuvant CRT and adjuvant chemotherapy type was similar to that for SCC with 45 Gy and cisplatin as a combined strategy before the operation, and four cycles of cisplatin and vinorelbin after the operation procedure.

## Discussion

This case shows a rare constellation of a HPV-positive rectal SCC with metastasis in both ovaries in a HIV-negative patient. While there is a clear association of anal SCC with the most common subclasses of high risk HPVs, including types 16, 18, 31, and 33, a correlation to rectal SCC has not been clearly established yet [16, 17]. One possible explanation for HPV-associated SCC in the rectum is circulating HPV DNA as discussed by Ambrosio *et al.* [8]. An alternative pathogenetic pathway may be a misplacement of HPV-infected neoplastic epithelial cells from the anal canal with subsequent implantation of these tumor cells into the rectal mucosa.

Primary SCC of the rectum is rare with an incidence rate of 0.1–0.25 per 1000 cases of colorectal cancers [2, 4–6]. These tumors appear more often in women than in men (66% versus 34%) and mostly in the fifth and sixth decades of life; the mean age is 57, the published age range is 39–93 [18].

Symptoms of rectal SCC are similar to those of adenocarcinoma with abdominal or pelvic pain, anal bleeding, changes in bowel habits. and weight loss.

The pathophysiology of rectal SCC is controversial. Chronic irritation after radiation, gastrointestinal infections with *Entamoeba histolytica*, and schistosomiasis are described as reasons for squamous metaplasia which may lead to malignant progression [12, 19–21]. Pluripotent stem cells in gastrointestinal mucosa are suggested by Ouban *et al.* as an origin of colorectal SCC [22]. Furthermore, a squamous differentiation of adenoma in cloacogenic polyps was described [23].

Already, in 1979, Williams *et al*. suggested some criteria that are necessary for the diagnosis of a primary colorectal SCC: no evidence of SCC of any other origin, no extension from the anal squamous epithelium, and absence of squamous-lined fistula [12, 24].

The diagnostic work-up should include a CT scan, pelvic MRI, colonoscopy with tissue biopsy, and endosonography. In cases of rectal SCC a PET-CT scan is recommended.

Metastasis of colorectal malignancies (mostly adenocarcinoma) in the ovaries is rare but well described in the literature and should always be considered in cases of cystic formations on preoperative imaging. In the absence of foreign metastasis and suspicious lesions in ovaries only, an adnexectomy should be performed for diagnostic reasons on the one hand and tumor reduction on the other hand.

In our case, there were tumors in both ovaries and a relatively small tumor of the rectum posing the question of the original primary site of the tumor. All the lesions were positive for HPV-16. The first interpretation was that of a SCC derived from a mature teratoma of the ovaries with metastasis to the rectum, which is well described in the literature. However, an association to HPV is rare and mentioned only once in the literature as yet [25]. Retrospective analyses of all tissue samples including immunohistochemistry and HPV *in situ* hybridization showed that both the rectal and the ovarian squamous carcinoma cells were positive for p16 and HPV of the high risk group. By contrast, the glandular ovarian epithelial cells were HPV-negative and were reclassified as foci of extensive endosalpingiosis (Fig. 4a–d). Molecular HPV genotyping revealed HPV-16 in all tumorous lesions of our patient (rectum, ovaries, and AIN) and a final diagnosis of an HPV-associated rectal squamous cells carcinoma with double-sided metastasis to the ovaries was rendered.

Surgery is the recommended course of treatment for primary colorectal SCC. In cases of ovarian teratomas with malignant transformation, hysterectomy can improve overall survival and was performed in our case [26]. A preoperative combined CRT can be performed in rectal SCC for improved local disease control. Which surgical strategy is chosen depends on the distance of the tumor to the anal verge; however, it should not differ from colorectal adenocarcinoma.

The follow-up should include a CT scan, pelvic MRI, and colonoscopy. In cases of colorectal adenocarcinoma, a PET-CT scan is not necessary [27]. However, in cases of colorectal SCC a PET-CT scan for primary diagnostics is recommended to rule out a metastasis at another location and is also recommended for follow-up.

Data about the prognosis of primary colorectal SCC are sparse. The overall 5-year survival rate is estimated at 32% with significant variation by stage [24]. An improved outcome is suggested with platinum-based CRT [1, 28].

This case shows a rare constellation with metastasis of rectal SCC in both ovaries. The importance of the guidelines which Williams *et al.* established is illustrated in this case [12]. Moreover, it shows the importance of the treatment of metastasis in colorectal SCC [12].

The role of adjuvant HPV vaccination is controversial. The data in the literature are insufficient. Some data suggest a decreased recurrence rate after adjuvant vaccination [29, 30]. With the implementation of HPV vaccinations we may observe a decrease in SCC at any location in the future. Furthermore, HPV vaccination should be recommended after HPV-associated diseases [31].

## Conclusion

Colorectal SCCs are rare. In such cases, metastatic disease at any other location should necessarily be excluded. A PET scan can be a helpful diagnostic tool and should be recommended. Discussion about therapeutic strategy should be interdisciplinary and include CRT and surgery, especially if the rectum is involved. The importance of the treatment of oligometastatic disease in colorectal cancer is clearly shown in this case.

## Data Availability

All data generated or analyzed during this study are included in this published article.

## Abbreviations

AIN: Anal intraepithelial neoplasia
CA: Cancer antigen
CEA: Carcinoembryonic antigen
CRT: Chemoradiation therapy
CT: Computed tomography
HPV: Human papillomavirus
PET: Positron emission tomography
SCC: Squamous cell carcinoma
TME: Total mesorectal excision
